# Psychosis to Pancreatitis: A Case Study Exploring the Risks of Hypertriglyceridemia in a Patient Treated With Olanzapine

**DOI:** 10.7759/cureus.70585

**Published:** 2024-10-01

**Authors:** Eduardo D Espiridion, Andrew S Murdock, Lorenzo E Guani, Angelica Arshoun

**Affiliations:** 1 Psychiatry, Drexel University College of Medicine, Philadelphia, USA; 2 Psychiatry, Reading Hospital, Tower Health, West Reading, USA

**Keywords:** diabetes, hypertriglyceridemia induced pancreatitis, lipids, psychosis, severe pancreatitis

## Abstract

Olanzapine is an antipsychotic medication that is used in the management of schizophrenia and bipolar disorder, but it is not without any adverse effects. We present the following case of a 24-year-old man with a history of schizoaffective disorder, obesity, and anxiety, who developed hypertriglyceridemia-induced acute pancreatitis after six months on olanzapine. Despite his adherence to the medication, routine metabolic monitoring was not performed leading to a delayed diagnosis of hypertriglyceridemia and subsequent complications. The case underscores the critical need for regular metabolic monitoring in patients prescribed olanzapine to prevent severe adverse effects and guide timely intervention. Enhanced adherence to monitoring guidelines and consideration of alternative treatments may help mitigate such risks.

## Introduction

Olanzapine, a second-generation antipsychotic, is indicated for patients with acute mania or as a maintenance treatment for patients with schizophrenia and bipolar disorder [1,2]. According to the Diagnostic and Statistical Manual of Mental Disorders, Fifth Edition, Text Revision (DSM-5-TR), to meet the criteria for the diagnosis of schizophrenia, the patient must have experienced at least two of the following symptoms: delusions, hallucinations, disorganized speech, disorganized behaviors, and negative symptoms. Bipolar disorder includes symptoms of mood lability, grandiosity, decreased need for sleep, racing thoughts, pressured speech, poor impulse control, and depression. Its mechanism involves antagonism of the D2 dopamine receptors with reduced off-target activity compared to first-generation antipsychotics [2]. Despite its benefits, olanzapine has an increased risk of metabolic syndrome compared to other second-generation antipsychotics [3]. As a result, current guidelines recommend that patients started on these medications have their body mass index (BMI), blood pressure, and metabolic laboratories, such as fasting plasma glucose, hemoglobin A1c, and lipids, checked at four weeks, eight weeks, 12 weeks, six months, and one year and then annually after initiating treatment to monitor for metabolic derangements [3]. An increasing number of reports have shown that olanzapine can cause hypertriglyceridemia-induced acute pancreatitis [4]. Our case study illustrates a patient on olanzapine who did not have routine bloodwork and remained on the medication despite increasing metabolic disturbances. This resulted in pancreatitis and a long hospital stay. The aim of this report is to emphasize the importance of routine lab testing and illustrate an extreme complication that can develop when using olanzapine. 

## Case presentation

This is a 24-year-old male with a past medical history of schizoaffective disorder, obesity, attention-deficit hyperactivity disorder (ADHD), and anxiety who presented to the emergency room of a community hospital with progressively worsening abdominal pain associated with nausea and vomiting. On physical exam, the patient was obese with a BMI of 33 kg/m^2^, afebrile, and tachycardic and had diffuse abdominal pain without rebound or guarding. The mental status exam was notable for flat affect, and he denied depressive or psychotic symptoms. Labs on admission were significant for a triglyceride level of 8,320 mg/dL with a total cholesterol level greater than 618 mg/dL, a lipase level of 3500 IU/L, a glucose level of 296 mg/dL with a hemoglobin A1c of 8.2%, and a β-hydroxybutyrate level of 1.42 mmol/L (Table 1).

**Table 1:** Significant laboratory findings on admission. AST: aspartate aminotransferase; ALT: alanine aminotransferase; pCO2: partial pressure of carbon dioxide; pO2: partial pressure of oxygen

Component	Result	Reference range
Sodium	135 mmol/L	136-145 mmol/L
Blood urea nitrogen	8 mg/dL	9-23 mg/dL
Creatinine	0.66 mg/dL	0.73-1.18 mg/dL
Glucose	296 mg/dL	70-100 mg/dL
Hemoglobin A1c	8.2%	<5.7%
Calcium	8.2 mg/dL	8.7-10.4 mg/dL
Magnesium	1.4 mg/dL	1.6-2.6 mg/dL
AST	60 IU/L	<34 IU/L
ALT	57 IU/L	10-49 IU/L
Lipase	3500 IU/L	12-53 IU/L
White blood cells	13.4 x 10^3^/uL	4.8-10.8 × 10^3^/uL
Hemoglobin	13.3 g/dL	12-16 g/dL
Blood pH	7.272	7.35-7.45
Blood pCO2	32.4 mmHg	35-48 mmHg
Blood pO2	79.1 mmHg	83-108 mmHg
β-Hydroxybutyrate	1.42 mmol/L	<0.27 mmol/L
Bicarbonate	14.9 mmol/L	21-28 mmol/L
Triglycerides	8,320 mg/dL	<150 mg/dL
Total cholesterol	>618 mg/dL	<200 mg/dL

**Figure 1:**
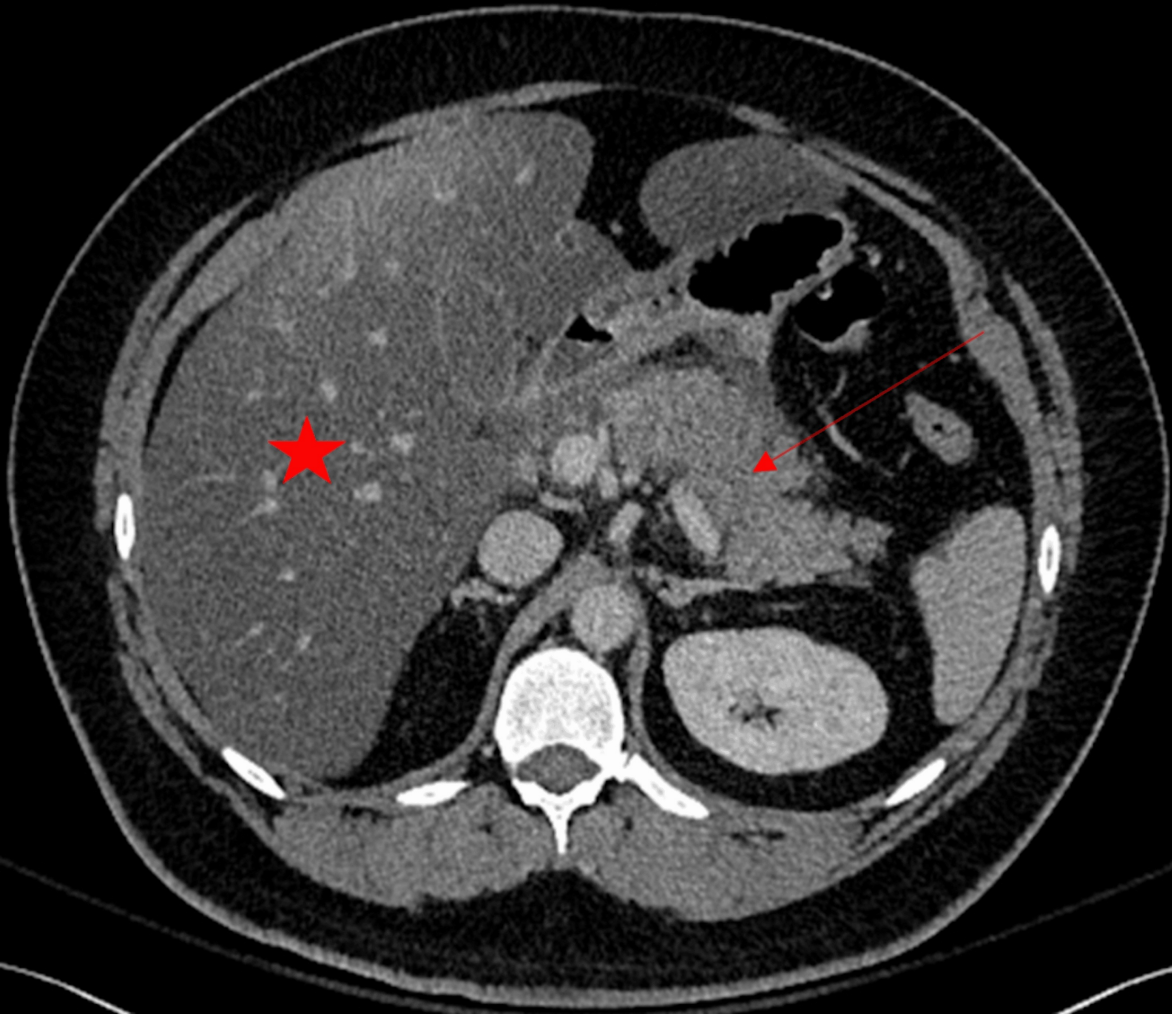
CT scan of the abdomen with contrast. The image demonstrates diffuse hepatic steatosis (star) and substantial fat stranding as well as fluid extending into the bilateral paracolic gutters (arrow). Fat stranding and fluid accumulation can extend into the bilateral paracolic gutters, consistent with findings in acute pancreatitis.

Abdominal and pelvic CT scans showed free fluid around the duodenum and pancreatic head with hepatic steatosis and hepatomegaly (Figure 1).

Additionally, it showed diffuse enlargement and inflammatory stranding surrounding the pancreas consistent with pancreatitis (Figure 1). The patient was diagnosed with triglyceride-induced acute pancreatitis and diabetic ketoacidosis requiring intravenous fluids, pain medication, and insulin therapy. 

Six months prior to his admission to the hospital, he was transitioned by an outpatient psychiatrist to 20 mg nightly olanzapine from risperidone. The patient's psychiatric symptoms reportedly worsened with command auditory hallucinations and sleeping difficulties. Additionally, he was taking fluoxetine 40 mg daily and trazodone 100 mg nightly. Since then, he reported being compliant with his medications; however, he noted that he had an excessive appetite and gained a significant amount of weight. His blood glucose and cholesterol levels were never measured or followed until this hospitalization. 

The patient was diagnosed with schizoaffective disorder four years prior to admission. His symptoms included sad mood, flat affect, auditory hallucinations, referential thoughts, and suicidal ideation. The patient met the criteria for both schizophrenia and bipolar disorder, and there was a period when he was psychotic with no mood symptoms. He had four previous inpatient psychiatric admissions. Family history was significant for hyperlipidemia, hypercholesterolemia, and various psychiatric disorders including bipolar disorder. Social history was significant for a diet consisting mainly of fast food and a multiyear history of vaping nicotine roughly one cartridge per week. 

On admission to the hospital, the patient's olanzapine was discontinued, as it was believed to be the etiology of his current presentation. Initially, his hypertriglyceridemia seemed to respond well to intravenous insulin with a decrease to 2,912 mg/dL. However, he was transferred to the intensive care unit (ICU) for refractory intravenous fluid resuscitation for his pancreatitis and had persistently elevated triglycerides on day 2 of his hospitalization. On the same day, he underwent plasmapheresis, which successfully lowered his triglycerides to 933 mg/dL. After several complications in his hospitalization, including acute kidney injury, acute respiratory distress syndrome, and hospital-acquired pneumonia, he was started on atorvastatin 40 mg nightly and fenofibrate 200 mg daily on day 4 of his hospital stay. He was discharged on day 13, and his triglyceride level was 335 mg/dL. On discharge, he was prescribed lurasidone 40 mg daily while he awaited follow-up with an outpatient psychiatric center and his primary care provider. 

## Discussion

While second-generation antipsychotics have a safer adverse effect profile than the first-generation class, they still act on multiple off-target receptors including serotonergic, alpha-1 adrenergic, histaminergic, and muscarinic receptors. They tend to be less associated with extrapyramidal symptoms [2]. The activation of sterol regulatory element-binding protein 1 (SREBP-1c) and neurotransmitter signaling via dopamine, gamma-aminobutyric acid (GABA), and serotonin are implicated in the development of various metabolic derangements [5]. In as short as six months, olanzapine, like other second-generation antipsychotics, can cause hepatic steatosis, weight gain, and metabolic derangements, including induced insulin resistance and dyslipidemia [6]. A meta-analysis has shown that triglycerides, total cholesterol, and low-density lipoprotein cholesterol (LDL-C) can increase in patients as early as week 4 of olanzapine treatment [6]. Elevated lipids can damage different organ systems, such as the cardiovascular system, leading to increased mortality [5]. 

This case adds to the increasing evidence that hypertriglyceridemia-induced pancreatitis is a potentially life-threatening adverse effect of olanzapine [4]. It emphasizes the importance of routine lipid panels when starting a new antipsychotic medication [2]. Had this patient received adequate laboratory testing, his new-onset diabetes mellitus and elevated lipid levels might have been detected allowing providers the opportunity to adjust his medication regimen and potentially prevent the development of his pancreatitis. 

The exact mechanism by which olanzapine causes insulin resistance and other metabolic symptoms is poorly understood. Multiple studies, some of which were compiled in a meta-analysis by Li et al., have proposed pathways implicated in olanzapine-induced metabolic derangements [6]. A study with mouse models suggests that chronic olanzapine use induces insulin resistance via inflammatory reactions in peripheral adipose tissue [7]. The cytokines released, interleukin-1 beta (IL-1β), IL-6, IL-8, and tumor necrosis factor alpha (TNF-α), appear to suppress inhibitor of nuclear factor kappa B (IκBα) leading to metabolic symptoms via the activation of the nuclear factor kappa B (NF-κB) pathway [7]. Insulin resistance can then lead to lipoprotein lipase inhibition and SREBP-1c stimulation causing increased LDL-C and triglyceride levels, respectively [8,9]. These pathways, coupled with the disrupted genetic regulation of lipid metabolism, cause enhanced lipogenesis in the liver and decrease lipid clearance from the bloodstream [10,11]. Additionally, worsening glycemic control has a strong association with an increased risk of acute pancreatitis [12]. Dyslipidemia might also be secondary to weight gain caused by increased food intake [6]. The antagonism of serotonin and histamine H1 receptors in the hypothalamus and increased levels of leptin and ghrelin have been suggested as reasons for increased food intake and subsequent weight gain in patients on olanzapine [13,14]. Ultimately, it is possible that metabolic derangements arise from the summation of multiple off-target pathways. 

Such metabolic derangements can lead to a variety of additional health concerns. Hypertriglyceridemia, for example, can trigger the development of acute pancreatitis. Various theories regarding its exact mechanism include increased plasma viscosity which might induce ischemia resulting in organ inflammation [7]. Other theories propose that increased levels of fatty acids lead to micelle formation that damages platelets and vascular endothelium [7]. Regardless of the mechanism, recent evidence shows an increased incidence of metabolic side effects in patients taking olanzapine [5]. These side effects, as demonstrated by our report, can be life-threatening necessitating close monitoring and prompt interventions. 

Given the variety of downstream adverse events related to the side effects of olanzapine and other second-generation antipsychotics, the importance of regular monitoring cannot be understated. Current guidelines suggest monitoring patients regularly while on olanzapine [2]. The aforementioned assessment for metabolic complications includes BMI, blood glucose, lipid profile, and blood pressure at various time intervals. However, these guidelines have not been formally updated with consensus since 2017 [15]. Further, they do not account for other conditions that could increase a patient's risk for dyslipidemia, including pre-existing diabetes, excessive alcohol consumption, and genetic conditions, such as familial lipoprotein lipase deficiency [10]. Researchers have also found that adherence to these guidelines is suboptimal with less than 12% of patients undergoing lipid testing [16]. It is suggested that patients on antipsychotics with persistent dyslipidemia be switched to an antipsychotic with less metabolic effects, such as ziprasidone, lurasidone, or aripiprazole, or started on a lipid-lowering therapy [3,17]. 

In the future, health practitioners should continue to follow guidelines for the laboratory monitoring of patients on olanzapine and other antipsychotics. Future studies could explore the link between pancreatitis and olanzapine use in patients at increased risk for hypertriglyceridemia including those with a family history of hyperlipidemia, a vaping history, or an alcohol use disorder [10]. Research should also assess the risk of pancreatitis with other antipsychotic medications to determine if other second-generation antipsychotics have better risk profiles. Additional studies could investigate optimized lipid-managing treatments should patients need to remain on olanzapine. 

## Conclusions

A balance must be struck between treating psychiatric conditions and managing hyperlipidemia and other adverse effects of antipsychotics. Routine monitoring would allow providers to make appropriate medication modifications to manage symptoms while reducing potentially severe adverse effects and polypharmacy. Ultimately, this patient had a two-week hospital stay due to complications that could have been prevented with routine laboratory monitoring and medication adjustments. 
